# Fluorodeoxyglucose positron emission tomography may aid the diagnosis of aggressive primary prostate cancer: A case series study

**DOI:** 10.3892/ol.2013.1747

**Published:** 2013-12-09

**Authors:** RICCARDO BARTOLETTI, ENRICO MELIANI, ANDREA BONGINI, CARLO MAGNO, TOMMASO CAI

**Affiliations:** 1 Department of Medical and Surgical Critical Care, Urology Unit, University of Florence, Florence, Italy; 2 Urology Unit, Santa Maria Annunziata Hospital, Florence, Italy; 3 Department of Urology, University of Messina, Messina, Italy; 3 Department of Urology, Santa Chiara Regional Hospital, Trento, Italy

**Keywords:** prostate cancer, cancer metabolism, positron emission tomography, high-risk prostate cancer, prostate specific antigen

## Abstract

Recent evidence has shown that positive results may be observed for fluorodeoxyglucose-positron emission tomography (FDG-PET) in undifferentiated, biologically aggressive and metastatic tumors. The present study describes a case series of six patients with normal prostate-specific antigen (PSA) serum levels who underwent FDG-PET due to other causes. Positive PET results were observed at the prostate and the patients were subsequently diagnosed with high-risk prostate cancer. Clinical, anamnestic, laboratory and instrumental data were collected from six asymptomatic patients with total serum PSA levels of <4 ng/ml who had undergone FDG-PET due to other causes. The FDG-PET and prostate biopsy were positive for prostate cancer. All the patients were treated with radical intent. The median age was 66 years (range, 52–72 years), the median total PSA value was 2.4 ng/ml (range, 1.5–3.9 ng/ml) and the body mass index was 26.4 (range, 21.8–30.2). Three of the six patients underwent FDG-PET due to a clinical suspicion of multiple myeloma, while three patients were examined for other oncological diseases. The pathological analysis at the prostate biopsy revealed three patients with a Gleason score of 6, two with a score of 7 (4+3) and one with a score of 8 (4+4). Five of the six patients were treated by radical prostatectomy and one by radiotherapy. The pathological analysis revealed one patient of pT2a stage, three of pT2c and one of pT3b. No patients demonstrated lymph node invasion. The definitive Gleason score was 3+3 in one patient, 4+3 in one patient, 4+4 in two patients and 5+3 in one patient. Following a median follow-up time of six months (range, 1–12 months), five of the six patients underwent FDG-PET again, which revealed negative results. At the end of this study, these patients were alive without evidence of disease. By contrast, one patient demonstrated positive FDG-PET results. In conclusion, FDG-PET has been used to characterize prostate cancers in patients with apparently normal PSA levels.

## Introduction

A raised prostate specific antigen (PSA) level is the first sign of prostate cancer in the majority of asymptomatic patients, although subjects with high-risk disease may exhibit PSA levels within the normal range (1). These patients are only diagnosed early in cases with unexpected evidence of urinary symptoms and/or prostate nodules that are identified during a digital rectal examination. More frequently, patients with PSA levels within the normal range are diagnosed with prostate cancer later, in association with the presence of symptoms due to tumor spread or metastatic disease. Of these patients, few are subsequently clinically evaluated and treated using definitive treatments with curative intent (2). The clinical evaluation for tumor staging includes pelvic nuclear magnetic resonance (NMR), computed tomography (CT) and total bone scans, while choline positron emission tomography (PET) is currently used to investigate the presence of distant metastases or locally advanced tumor spread. PET [^11^C]- and [^18^F]-choline derivatives have also been successfully used to monitor patients following surgery, radiotherapy or hormonal treatments. However, false negative results have been previously reported. The use of choline for prostate cancer imaging is based on increased phosphorylcholine levels and elevated phosphatidylcholine turnover in prostate cancer cells. choline PET has also been previously evaluated in the early detection of prostate cancer with conflicting results (3–7). By contrast, PET and PET-CT with fluorodeoxyglucose (FDG) have demonstrated a limited sensitivity for prostate cancer detection, but may easily identify a positive result in undifferentiated, biologically aggressive and metastatic tumors (8). A recent study by Minamimoto *et al*(9) investigated the FDG-PET screening cancer program using 155,456 subjects and identified a 37.0% PET sensitivity in patients with prostate cancer. To date, a low importance has been assigned to FDG-PET to investigate the potential identification of patients with aggressive primary prostate cancer among subjects with low PSA levels. The present study reports a case series of six patients with normal PSA serum levels, who underwent FDG-PET due to other causes. The PET results were positive at the prostate and the patients were subsequently diagnosed with high-risk prostate cancer. Written informed consent was obtained from the patients.

## Case reports

### Case 1

A 61-year-old patient presented with no significant urinary symptoms, a total PSA serum level of 3.9 ng/ml and a body mass index (BMI) of 24.4. The patient was suspected of having multiple myeloma due to the presence of osteolytic areas on the skull, which were observed on an X-ray following a consultation with the otorhinolaryngologist due to a chronic nasal obstruction, asthenia, slight anaemia and a headache. FDG-PET was suggested by the hematologist and was identified to be positive at the prostate and oropharynx. The digital rectal examination (DRE) was negative for nodules and prostate cancer was not suspected. An 18-core prostate biopsy was then performed resulting in five bilateral positive cores and a pathological diagnosis of prostate adenocarcinoma (Gleason score 6; 3+3). The total bone scan was negative. The patient underwent a retropubic radical prostatectomy with pelvic limphadenectomy. The pathological evaluation demonstrated pT2cN0 prostate adenocarcinoma involving the two lobes and the right apex (Gleason score 8; 5+3), with negative margins. The total PSA levels were 0.05 and 0.12 ng/ml at the three- and six-month follow-up appointments, respectively. Adjuvant conformational radiotherapy was then administered with a cumulative dose of 70 Gy and completed just a month later. At the six-month follow-up appointment*,* the PSA value had increased to 0.8 ng/ml and the PET-FDG result was positive at the prostatic fossa, which was the single remaining location of the disease.

### Case 2

A 72-year-old patient presented with no significant urinary symptoms and a BMI of 21.8. The total PSA serum level at the time of diagnosis was 3.27 ng/ml. Asthenia and back pain were investigated by spinal plain X-rays. The total bone scan was negative. An early suspicion of multiple myeloma indicated the requirement for FDG-PET, which was positive at the prostate gland. The DRE was negative for nodules or prostate indurations. The 18-core prostate biopsy was positive for prostate adenocarcinoma in two cores on the left lobe (Gleason score 6; 3+3). The patient was administered radiotherapy with a cumulative dose of 70 Gy. The total PSA level was 0.3 ng/ml following six months of the treatment. The final FDG-PET result was negative.

### Case 3

A 70-year-old patient presented with no urinary clinical symptoms and a BMI of 23.6. The total PSA level was 2.7 ng/ml. The patient had already been diagnosed with myasthenia gravis, muscle weakness and fatigue. FDG-PET was performed to investigate the possible presence of a thymoma, but instead revealed a positive result at the prostate gland. The 18-core prostate biopsy revealed the presence of a prostatic adenocarcinoma on the left lobe (Gleason score 7; 4+3). The patient underwent a radical retropubic prostatectomy and the pathological stage was classified as pT2cN0. The PSA level was 0.02 at 6 months post-surgery. The final FDG-PET result was negative.

### Case 4

**Figure 1:**
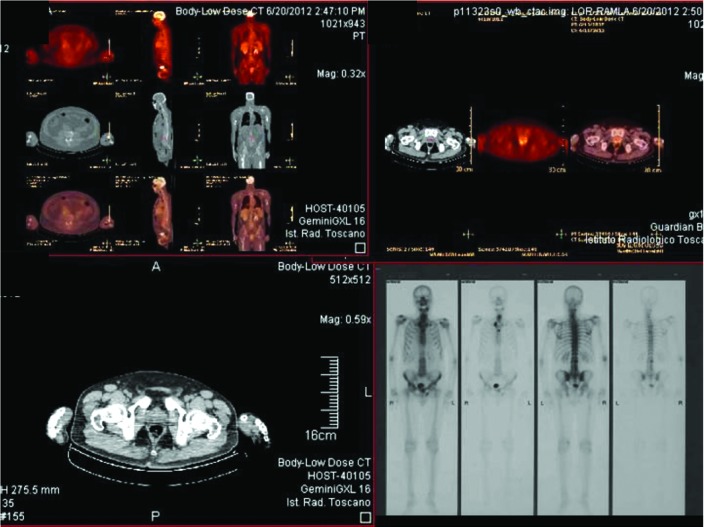
(^18^F-FDG-3′5MBq) fluorodeoxyglucose-positron emission tomography (FDG-PET) of case 4. FGD-PET prostate positive signaling was the only sign of prostate cancer that was identified during medical investigations due to other causes.

A 52-year-old patient with a BMI of 30.2 presented with asthenia and back pain, which were later confirmed as the first non-specific signs of prostate cancer. FDG-PET was performed due to a suspected case of multiple myeloma, and a positive signal was revealed at the prostate (Fig. 1). The PSA level was 2.0 ng/ml and the DRE was negative. An 18-core random prostate biopsy was positive for prostate cancer on the left lobe (Gleason score 6; 3+3). The bone scan and NMR transrectal coil imaging were negative. The patient underwent a retropubic radical prostatectomy. The pathological stage was classified as pT2aN0 (Gleason score 6; 3+3). No clinical signs or symptoms of disease were present at one month post-surgery.

### Case 5

A 64-year-old patient with a BMI of 28.5 presented with no pain or urinary symptoms. A hyperplastic and painful cervical lymph node was investigated using FDG-PET, which revealed a positive signal at the prostate. The PSA level was 2.18 ng/ml and the DRE was negative. The patient was diagnosed with prostate cancer following a 12-core prostate biopsy with cancer diffusion to the two lobes (Gleason score 7; 4+3) and subsequently underwent a retropubic radical prostatectomy. The pathological stage was classified as pT2cN0 (Gleason score 8; 4+4). The patient was observed to have no evidence of disease at a follow-up appointment, 7 months after surgery (PSA level, <0.01 ng/ml). The final FDG-PET result was negative.

### Case 6

A 68-year-old patient with a BMI of 29.1 presented with no pain or urinary symptoms. The patient had already been diagnosed with and treated for systemic non-Hodgkin lymphoma and now periodically underwent clinical and imaging monitoring. The FDG-PET identified a positive signal at the prostate, with a PSA level of 1.56 ng/ml and a negative DRE. Prostate cancer was diagnosed following a 12-core prostate biopsy with bilateral cancer diffusion (Gleason score 8; 4+4). The patient underwent a retropubic radical prostatectomy. The pathological stage was classified as pT3bN0 (Gleason total score 8; 4+4). The PSA level was <0.01 after 12 months and the final FDG-PET was negative. No apparent recurrences were observed for non-Hodgkin lymphoma or prostate cancer.

### Overall results

The pre-biopsy PSA levels of the six cases ranged from 1.56 to 3.9 ng/ml, with a median value of 2.4 ng/ml. The pathological evaluation demonstrated high-risk prostate cancer diagnoses in four of the five cases that underwent radical prostatectomies, while the one patient who was administered external beam radiotherapy was diagnosed with low-risk prostate cancer on the basis of the pathological analysis of the prostate biopsy. The disease staging was performed by NMR phased array and total bone scanning in all the patients without significant results in terms of primary tumor or tumor metastases identification. Clinical organ-confined prostate cancer was identified in all the cases on the basis of positive prostate core biopsies and positive FDG-PET signaling. Five patients, all <70 years old, underwent radical prostatectomies and one patient, who was 72 years old, was administered radiotherapy. The pathological investigations evidenced high-risk prostate cancer in four of the five patients that were treated by surgery (Gleason scores 7–10), one of whom had previously been diagnosed with low-risk disease. Four patients were observed to have organ-confined diseases (three pT2c and one pT2a; Gleason scores 5+3, 4+3, 3+3 and 4+4, respectively) and one patient exhibited extra capsular disease (pT3b Gleason score 4+4). All the cases that were treated by surgery had negative surgical margins. The post-operative PSA levels remained at <0.02 ng/ml in three cases at a mean eight-month follow-up time, but increased to 0.12 ng/ml following five months in the last case. The patient who was >70 years old and was previously treated with radiotherapy (Gleason score 3+3) at prostate biopsy, revealed a PSA level of 0.3 ng/ml following three months with stable disease (Table I). All the patients displayed negative FDG-PET signaling at the six months (range, 1–12 months) follow-up.

## Discussion

This is the first series of patients with apparently normal PSA levels, positive FDG-PET signaling and subsequent diagnoses of high-risk prostate cancer. Patients with PSA levels within the normal range of 0–4 ng/ml are usually only diagnosed with prostate cancer in cases of concomitant urinary and/or pain symptoms that are associated with the local extent and metastatic diffusion of the disease. Data obtained from the prostate cancer prevention trial (PCPT) indicates that up to 15% of males with a normal screening test (PSA level <4 ng/ml and negative digital rectal examination) have biopsy-detectable prostate cancer (10). By contrast, PSA screening is associated with substantial unfavorable effects. In the European Randomized Study of Screening for Prostate Cancer (ERSPC) screening group, the cumulative incidence was 7.4% compared with 5.1% in the control group, and over-diagnosed cancers were frequently treated with higher risks of adverse events (11,12). Due to these reasons, PSA level determination may be considered as an independent factor in the early diagnosis of prostate cancer. choline PET and PET/computed tomography (CT) have been scarcely used in the primary diagnosis of prostate cancer and with conflicting results, but they are currently used for the disease staging and monitoring of patients following treatment (8). Testa *et al*(13) compared the diagnostic performance of magnetic resonance imaging (MRI), 3-dimensional magnetic resonance spectroscopy (MRS), combined MRI and MRS and [^11^C]-choline PET/CT for imaging primary prostate cancer. The sensitivity of choline PET/CT was observed to be lower (55%) in comparison with MRS alone (81%) or MRS in combination with NMR (88%). By contrast, Yamaguchi *et al*(14) identified a higher sensitivity of choline PET (100%) compared with MRI alone (60%) or in combination with MRS (65%). The mean PSA values were 13.91 (range, 2.5–70) and 23.4 (range, 4.3–93.9) in the two series, respectively. Two patients with prostate cancer and normal PSA serum levels of 2.5 and 3.1 ng/ml at diagnosis, respectively, were included in the series by Testa *et al*(13), but advanced pathological stage (pT3a) and high-risk disease (Gleason score 4+3) was identified in one patient without specific information with regard to the sensitivity to choline PET imaging. Although a relatively low FDG uptake has been previously attributed to prostate cancer due to its slow metabolic rate and lower expression of glucose transport proteins, Hwang *et al*(15) analyzed FDG-PET/CT images from 12,037 subjects showing abnormal hypermetabolism in the prostate. A total of 120 patients were observed to exhibit abnormal FDG-PET/CT signaling, but 38 of these were subsequently investigated by prostate biopsy as a consequence of an abnormal total PSA serum level determination and/or the clinical suspicion of cancer at DRE (15). Of the 38 patients, 23 were confirmed to have prostate cancer with a median PSA level of 49.7 ng/ml. The cumulative results indicated that hypermetabolism in the prostate was incidentally detected in 1.5% of the patients, but only 65.2% underwent further clinical evaluation by DRE and/or serum PSA level determination (15). No data on patients with apparently normal PSA levels were thus collected (15). All the cases that were included in the present series had no urinary symptoms or clinical suspicions of prostate cancer and had normal PSA levels. Thus, the clinical diagnosis of prostate cancer was incidental. In the course of an FDG-PET cancer screening program in Japan based on a four-year nationwide survey conducted on 155,456 asymptomatic subjects, Minamimoto *et al*(9) identified positive findings indicating possible cancer in 16,955 cases (10.9%). The overall number of cancers that were actually detected was 1,912, but prostate cancer was identified in 165 patients, with a FDG-PET sensitivity of 37.0% (9). It may be concluded that approximately one-third of subjects with prostate cancer with positive FDG-PET findings may also have altered PSA levels. Decreased PSA levels in prostate cancer patients may have confounding factors, including the concomitant use of medical therapies, such as hormones, statins and non-steroidal anti-inflammatory drugs, and/or a higher BMI. Wright *et al*(16) observed that an increased BMI (<25, 25–29 and >30) was proportional to a decline in the geometric PSA values (1.18, 1.13 and 0.94, respectively) and hat obese males had lower age-adjusted PSA levels compared with males of a normal weight. The patients that were enrolled in the study had no previous medical treatments, none were obese and the BMI was <25, with the exception of one case. Thus, the PSA values may be considered to have been homogeneously measured. Helfand *et al*(17) identified that a panel of 17 risk alleles and a family history of prostate cancer is associated with an increased risk of the disease, even amongst patients with normal PSA levels and DRE. These data were not confirmed by the results that were obtained in the present series, as none of the patients that were investigated had a family history of prostate cancer. A recent study by Koochekpour *et al*(18) demonstrated a possible characterization of prostate cancer tumor cells in association with their metabolism. The metabolism of aggressive cancers may be altered by the presence of glutamate receptor 1 (GRM1) antagonists with subsequent development of the Warburg effect in the case of tumor-related induced hypoxia. Koochekpour *et al*(18) observed significantly higher serum glutamate levels in tumors with a Gleason score of >8 than in those with a Gleason score of <7 among African-Americans compared with Caucasian Americans. Glutamate metabolism alterations may represent a justification of possible positive FDG-PET signaling in patients with aggressive prostate cancer and a biomarker to be used in the future to characterize potentially evolutive or indolent prostate cancers. The main limitation of the present study consists of the non-consecutive patient selection that was due to the incidental diagnosis of prostate cancer in response to apparently normal PSA values. In all these cases, the total PSA measurement was not functional as a prognostic variable for monitoring the patients, while FDG-PET was imperative to determine the evidence of possible metastatic sites.

In conclusion, FDG-PET has been used to define the clinical suspicion for prostate cancer in a small series of asymptomatic patients with normal PSA levels. This phenomenon may be justified with a strong correlation between the metabolism of tumor cells and mitochondrial activity due to the Warburg effect. FDG-PET may be considered for future studies in order to characterize the aggressive behavior of primary prostate cancers in patients with normal PSA levels.

## Figures and Tables

**Table I:** Patient characteristics and medical histories.

Case	Age, years	BMI	Cause of clinical suspicion FDG-PET	Site of positive FDG-PET signaling	PSA, ng/ml	Biopsy	Clinical stage	Treatment	Pathological stage	Pathological Gleason score	Follow-up, months	Final status
1	61	24.47	Suspicion of myeloma	Prostate and oropharynx	3.90	3+3 bilateral	T0	RP	pT2cN0	5+3	6	Increased PSA, negative FDG-PET
2	72	21.80	Suspicion of myeloma	Prostate	3.27	3+3 left lobe	T0	RT	ND		6	PSA<0.02, negative FDG-PET
3	70	23.66	Myasthenia	Prostate	2.70	4+3 left lobe	T0	RP	pT2cN0	4+3	6	PSA<0.01, negative FDG-PET
4	52	30.20	Suspicion of myeloma	Prostate	2.00	3+3 left lobe	T0	RP	pT2aN0	3+3	1	Not yet evaluated
5	64	28.50	Latero cervical lymph node	Prostate	2.18	4+3 bilateral	T0	RP	pT2cNo	4+4	7	PSA<0.1, negative FDG-PET
6	68	29.10	Non-Hodgkin lymphoma	Prostate	1.56	4+4 bilateral	T0	RP	pT3bN0	4+4	12	PSA<0.02, negative FDG-PET

BMI, body mass index; FDG-PET, fluorodeoxyglucose-positron emission tomography; PSA, prostate-specific antigen; RP, radical prostatectomy; RT, radiotherapy; ND, not defined.
